# Ventriculoperitoneal shunt failures at Red Cross War Memorial Children’s Hospital

**DOI:** 10.1007/s00381-024-06466-w

**Published:** 2024-05-23

**Authors:** J. S. Lazarus, E. Ohonba, Y. J. Li, U. K. Rohlwink, A. A. Figaji, J. M. N. Enslin

**Affiliations:** 1 Department of Surgery, Division of Neurosurgery, University of Cape Town, Cape Town, South Africa; 2 Division of Neurosurgery, Red Cross War Memorial Children’s Hospital, Cape Town, South Africa; 3 Department of Cardiovascular Medicine, Radcliffe Department of Medicine, University of Oxford, Oxford, UK

**Keywords:** Ventriculoperitoneal shunt failures, Hydrocephalus, Shunt sepsis

## Abstract

**Introduction:**

Ventriculoperitoneal shunt (VP shunt) insertion is one of the mainstays of treatment of hydrocephalus and although very effective, a high rate of shunt failure persists globally. The purpose of the study was to quantify the ventriculoperitoneal shunt failure rate at Red Cross War Memorial Children’s Hospital (RCWMCH) and assess potential factors contributing to shunt failures.

**Methods:**

A retrospective review of VP shunts done at RCWMCH between August 2015 through December 2019 was performed. Operative notes, discharge summaries and patient folders were reviewed to collect information about patient age, aetiology of hydrocephalus, index vs revision shunt, shunt system and other noticeable variables. Overall shunt failure was recorded. Univariate and multivariate models were used to determine causal relationship.

**Results:**

Four hundred and ninety-four VP shunt operations were performed on 340 patients with 48.8% being index shunts and 51.2% revision shunts. The average patient age was 3.4 months. The total VP shunt failure rate over the study period was 31.2%, with a 7.3% infection rate, 13.6% blockage and 3.6% disconnection rate. The most common aetiologies were post-infectious hydrocephalus 29.4%, myelomeningocele 19.7% and premature intraventricular haemorrhage 14.1%. Orbis-sigma II (OSVII), distal slit valves and antibiotic-impregnated catheters were used most frequently. Failure rates were highest in the revision group, 34.7% compared to 27.3% in index shunts. Sixty-five percent (65%) of the head circumferences measured were above the + 3 Z score (> 90th centile).

**Conclusion:**

VP shunt failure occurs most commonly in revision surgery, and care should be taken at the index operation to reduce failure risk. Surgeon level, duration of surgery, aetiology of hydrocephalus and shunt system used did not influence overall failure rates. A closer look at larger head circumferences, their effect on shunt systems and the socio-economic factors behind late presentations should be investigated further in the future.

## Introduction

Hydrocephalus is a common paediatric condition faced by neurosurgeons [1]. A wide variety of aetiologies and theories behind its pathophysiology are postulated. If left untreated, hydrocephalus results in severe morbidity and mortality [2].

Since the first documented permanent ventricular shunt, placed by Mikulicz in 1893, cerebrospinal fluid (CSF) shunting has evolved and has since become the mainstay of treatment for hydrocephalus [3]. Shunt insertion is mentioned as one of only two neurosurgical procedures listed on the 44 essential surgeries published by the World Bank group [4, 5].

Sadly, CSF shunt complication rates are still high, reported as between 8.6 and 50% in developing countries [3]. Up to 30% of shunts will fail within 1 year of insertion [6]. Shunt failure is described as the need for any surgical procedure for definitive CSF diversion in a previously shunted patient [7]. The most common reasons are blockage, infection and shunt disconnection.

Various authors [8–10] have tried to identify risk factors for shunt failure and many [11–14] have studied factors linked to shunt survival. The aetiology of hydrocephalus, shunt type, surgeon experience and previous revisions have all been linked to shunt failure and poor outcomes.

There is an increased risk of shunt sepsis and failure every time a revision is needed; shunt failure is shown to even impact cognitive development in children [15].

Given the wide range of complication rates reported and the variation in hydrocephalus aetiology and patient demographics in different regions, it is important that each institution documents its own complication rates and shunt outcomes. This is also the basis for understanding how interventions or protocol changes influence shunt outcomes. With this study, we aimed to record our shunt insertion and failure rates for the first time at our institution. It will attempt to highlight certain risk factors that are specific to the population subgroup that contribute to shunt failure and the information to implement change. This may ultimately lead to a reduction in shunt failure rates and improve patient outcome.

## Methods

Following institutional review approval, a retrospective folder review of all patients undergoing a ventriculoperitoneal shunt insertion between August 2015 and December 2019 was conducted. This period was selected as departmental data collection protocols were amended prior to this. Patients under the age of 13 are treated at Red Cross War Memorial Children’s Hospital (RCWMCH), although not uncommonly, slightly older patients do present for emergency surgery.

We excluded, in isolation, ventriculopleural shunts, ventriculoatrial shunts, ventriculosubgaleal shunts, external ventricular drains (EVDs) and endoscopic third ventriculostomy (ETV).

Information was obtained from operative notes, discharge summaries and electronic radiological archiving systems. Outcome data was analysed from patient folders retrospectively. Data collected included patient age, sex, aetiology of hydrocephalus, time of shunt insertion and surgeon seniority. Each shunt insertion was designated as an index shunt (first shunt placed in patient) or a revision shunt (all subsequent shunts inserted), and the type of shunt was recorded. Additional factors, such as the use of an endoscope and the number of surgeons involved in the case, were also collected.

The aetiology of hydrocephalus was categorised as post-infectious/TB meningitis, myelomeningocele, posterior fossa/brainstem tumour, prematurity-related intraventricular haemorrhage, optic pathway glioma/hypothalamic glioma/ventricular tumours, aqueduct stenosis/pineal region tumour, cysts, cortical malformations, encephalocele, post-traumatic hydrocephalus, and in cases when no discernible cause was found, “other”. Head circumference was recorded as per the South African ‘Road to Health Chart’, using the Z-score classification. This chart is validated for the South African population.

The timing of surgery was classed as: during working hours, 07:00 to 17:00; after hours, 17:01 to 00:00; and after midnight, 00:00 to 06:59. The experience of the surgeon was classified as junior registrar (trainee, 2 or less years in the department), senior registrar (2 to 5 years in the department) and consultant.

Shunt failure was defined as ‘any reason for shunt to be revised/removed’. The cause (type) of shunt failure was as determined by the operating surgeon at the time of shunt insertion, revision or removal. In the minority of cases, a cause was not clear and labelled ‘not specified’. Reasons for shunt failure were classified as Infection; Blockage (proximal and distal obstruction grouped together); Shunt disconnection; Catheter malposition; Shunt erosion through the skin; Shunt fracture; Migration of proximal or distal catheter; CSF over drainage and Intraventricular haemorrhage. Due to low numbers in certain failure groups, subgroup clusters were made when analysing data.

Outcome data 2 years post-insertion of the last shunt was recorded, taking note of the patient’s last visit to RCWMCH. This was a convenient decision and allowed comparison to similar international studies. Shunt survival or subsequent revisions and the number of revisions were recorded.

### Statistical analysis

Measures of central tendency included median and interquartile or minimum-maximum range, given the non-parametric distribution of data. Frequency data are reported as number and percent, the association between shunt failure and potential risk factors was analysed using chi-square, Mann-Whitney’s *U* or Kruskal-Wallis tests using SPSS Statistical program (IBM)^®^. A *p*-value of < 0.05 was set as significant. Given the small sample size, multiple testing was not controlled for.

## Results

The demographic data of this study is summarised in Table 1. A total of 340 patients underwent 494 ventriculoperitoneal shunt surgeries during the study period. Head circumference (HC) was not routinely recorded in older patients (> 5 years old) and data was only available in 143 patients (42.7%). For the available data, the most common HC of patients was a + 3 z-score, with a 65% prevalence, as compared to the next highest of a 0 z-score of 20.9%.

**Table 1:** Patient demographics and aetiology of hydrocephalus (data are displayed as patient number (%))

Variable	Patients, *n* = 340 (%)
Age	
Min	1 day
Median	1 y 3 m
Max	16.6 yrs
Sex	
Female	154 (45.3)
Male	186 (54.7)
Head circumference (at presentation)	
3 z-score	93 (27.4)
2 z-score	10 (2.9)
1 z-score	2 (0.6)
0	30 (8.8)
−1 z-score	2 (0.6)
−2 z-score	4 (1.2)
−3 z-score	4 (1.2)
Not recorded	195 (57.3)
Aetiology of hydrocephalus	
Post-infectious/TB meningitis	100 (29.4)
Myelomeningocele	67 (19.7)
Posterior fossa/brainstem tumour	15 (4.4)
Premature intraventricular haemorrhage	48 (14.1)
Optic pathway glioma/hypothalamic tumour/intraventricular tumour/craniopharyngioma	18 (5.3)
Aqueduct stenosis/pineal region tumours/tectal tumours	15 (4.4)
Cysts	18 (5.3)
Cortical malformations	8 (2.4)
Encephalocele	4 (1.2)
Congenital	34 (10)
Other (suspected X-linked hydrocephalus/mucopolysaccharidosis/Down syndrome with CCJ instability/Crouzon’s syndrome/migrational disorder)	7 (2.1)
Post-traumatic	6 (1.8)

Post-infectious hydrocephalus, including TB meningitis (13.5%), was the most common aetiology of hydrocephalus at 29.4%, followed by spinal dysraphism (19.7%), most commonly myelomeningocele, and premature intraventricular haemorrhage (14.1%). The remaining aetiologies are shown in Table 1.

**Fig. 1:**
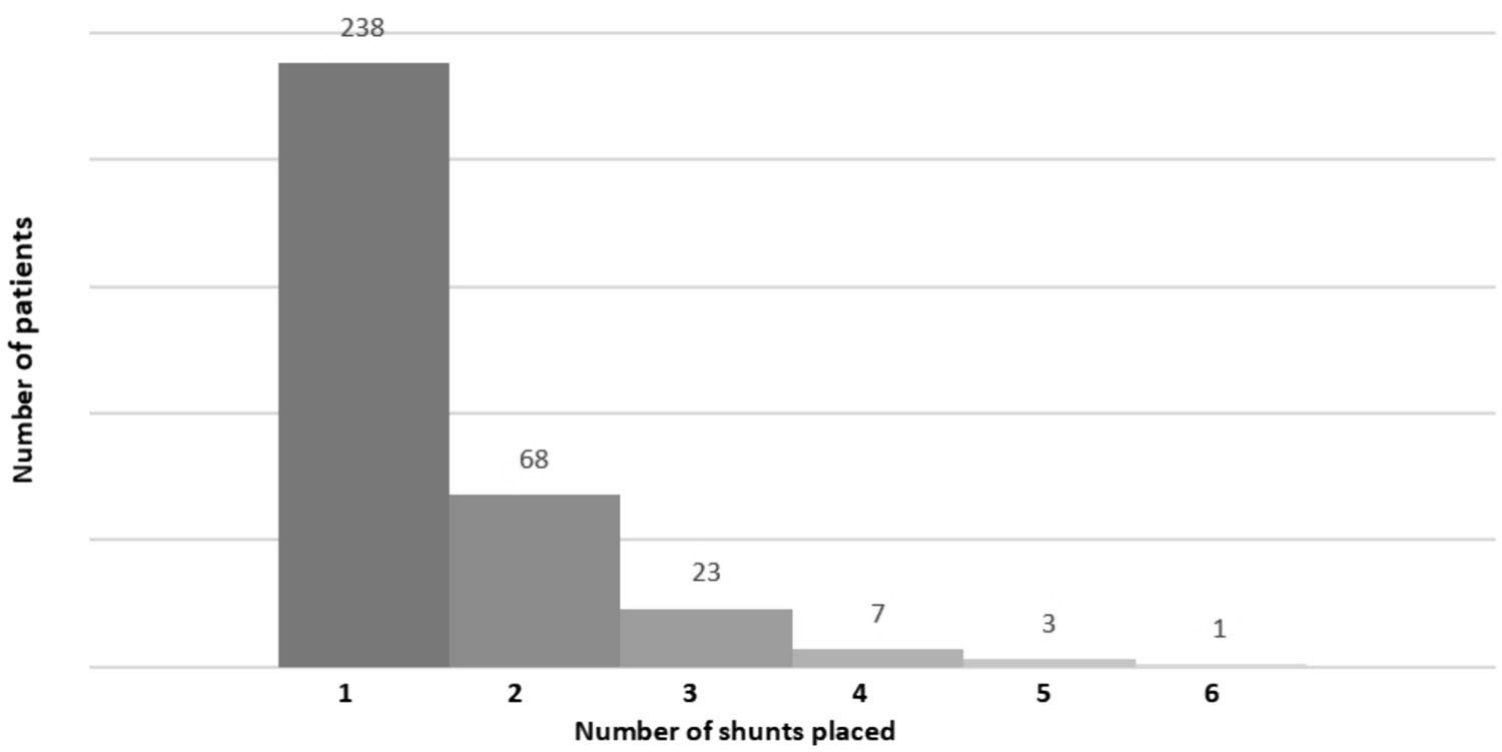
Total number of shunts placed per patient during the study period

Figure 1 shows the number of shunts placed per patient during the study period. One patient had a total of 6 shunts placed.

The overall failure rate for shunts inserted over the study period was 31.2%. The failure rate was higher in the revision group (34.75%) compared to the index group (27.3%) (*p* = *0.047*).

**Fig. 2:**
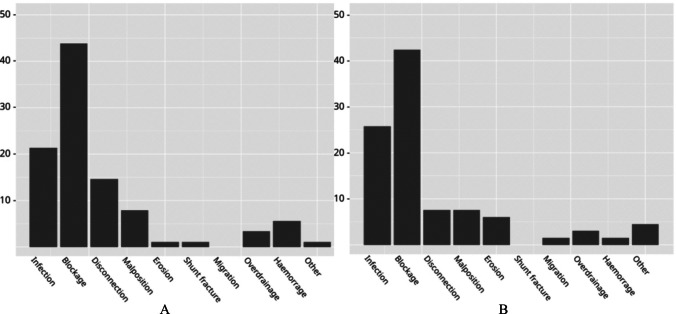
Bar graph illustrating types of shunt failures in Index (**A**) and Revision (**B**) groups as a percentage of total failures

**Fig. 3:**
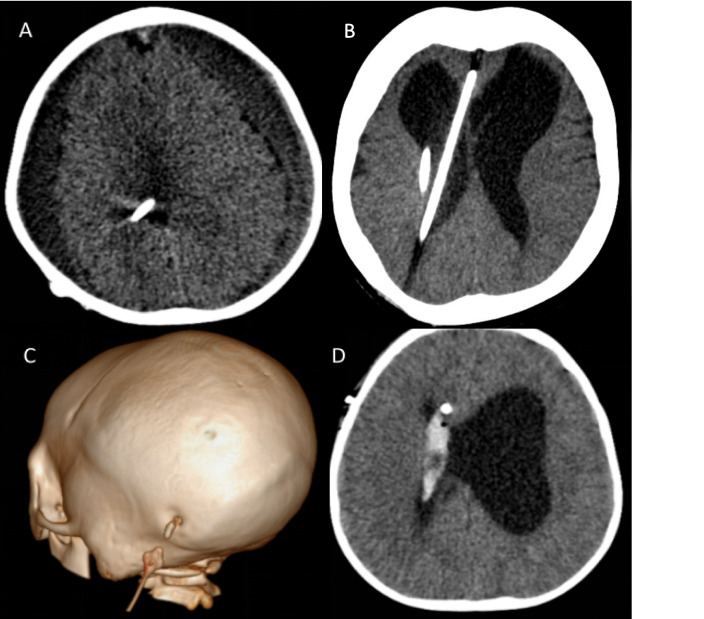
Figure showing various shunt complications encountered. **A** A patient with shunt overdrainage that developed bilateral chronic subdural haematomas. **B** Malpositioned ventricular catheter, with the catheter in the interhemispheric cistern, additional catheter in the right lateral ventricle. **C** Shunt disconnection with the distal catheter shown disconnected from the valve. **D** Intraventricular haemorrhage noted in right lateral ventricular following catheter placement

Figures 2 and 3 summarises and shows examples of aetiologies of shunt failures. Shunt blockage (proximal and distal obstruction) was the most common cause of shunt failure. Overall infection rates for the study (both index and revision) were 7.3%. The shunt disconnection rate was 3.6%, making this the third most common cause of failure. There was no increased risk of disconnection based on shunt type or level of surgeon. Shunt fracture was noted in 0.4%.

The time to failure was recorded as early (< 1 week post insertion), intermediate (1 week to 1 year) and late (> 1 year). No shunt infections occurred within a week of insertion, 91.7% occurred within the 1st year, and 8.3% occurred thereafter. No shunt disconnections occurred in the 1st week; 27.8% occurred after 1 year, with the remainder occurring in the intermediate period. Of note, malpositioned shunts failed during the 1st week in 41.7% and 50% failed in the intermediate range (Fig. 2).

None of the factors listed in Table 2 showed any significant correlation to shunt failures.

**Table 2:** Factors contributing to shunt failure

		**Index**					**Revision**				
		**Shunt failure**					**Shunt failure**				
		No		Yes		P value	No		Yes		P value
		No.	%	No.	%		No.	%	No.	%	
Age	< 6 months	94	39.0	42	17.4	0.504	26	10.3	15	5.9	0.651
	6 m to 1 year	26	10.8	6	2.5		18	7.1	6	2.4	
	1 year to 5 years	26	10.8	9	3.7		46	18.2	29	11.5	
	> 5 years	29	12.0	9	3.7		75	29.6	38	15.0	
Aetiology	Post-infectious/TB Meningitis	53	22.0	11	4.6	0.129	46	18.2	34	13.4	0.359
	MMC	36	14.9	15	6.2		32	12.6	14	5.5	
	Prem IVH	19	7.9	12	5.0		29	11.5	12	4.7	
	Other	67	27.8	28	11.6		58	22.9	28	11.1	
Level of Surgeon	Junior Registrar	105	43.6	38	15.8	0.886	93	36.8	48	19.0	0.956
	Senior Registrar	50	20.7	19	7.9		46	18.2	26	10.3	
	Consultants	20	8.3	9	3.7		26	10.3	14	5.5	
Surgical Time	< 50 min	83	35.2	22	9.3	0.056	53	21.5	29	11.7	0.899
	> 50 min	89	37.7	42	17.8		108	43.7	57	23.1	
Time of day	07:00–17:00	147	61.0	58	24.1	0.590	106	41.9	58	22.9	0.815
	17:01–00:00	21	8.7	7	2.9		41	16.2	19	7.5	
	00:01–07:00	7	2.9	1	0.4		18	7.1	11	4.3	
Shunt Used	Orbis-sigma II (OSVII)	56	23.2	32	13.3	0.095	82	32.4	52	20.6	0.200
	Distal Slit	74	30.7	23	9.5		31	12.3	12	4.7	
	Antibiotic impregnated shunt(AIS/ (Bactiseal®)	30	12.4	6	2.5		34	13.4	20	7.9	
	Other	15	6.2	5	2.1		18	7.1	4	1.6	
Endoscope used	Yes	19	7.9	5	2.1	0.448	17	6.7	8	3.2	0.758
	No	156	64.7	61	25.3		148	58.5	80	31.6	
CSF Protein	< 1	115	47.7	40	16.6	0.517	109	43.1	49	19.4	0.262
(g/L)	> 1	51	21.2	20	8.3		37	14.6	25	9.9	
	No result	8	3.3	6	2.5		19	7.5	14	5.5	
CSF Poly count	< 5	150	62.2	56	23.2	0.416	136	53.8	71	28.1	0.482
(/uL)	5 or more	16	6.6	4	1.7		17	6.7	7	2.8	
	No result	9	3.7	6	2.5		12	4.7	10	4.0	
CSF RBC count	< 100	87	36.1	32	13.3	0.113	94	37.2	47	18.6	0.528
(/uL)	100–1000	41	17	8	3.3		35	13.8	15	5.9	
	> 1001	39	16.2	20	8.3		24	9.5	16	6.3	
	Not recorded	8	3.3	6	2.5		12	4.7	10	4.0	

### Surgery duration and time of day

The mean surgical time was 59 min, with revision shunts taking longer (68 min) than index shunts (56 min). The shortest surgical time was 23 min and the longest was 180 min. Multiple surgeons resulted in faster surgical times (*p* = *0.002*) compared to single surgeon operations. Index shunt failure was seen more frequently if the surgical duration was longer than 50 min, with a *p*-value approaching significance (*p* = *0.056*).

The time-of-day that the surgery was performed was not significant neither for index nor revision shunts (*p* = *0.590* and *p* = *0.815*, respectively).

### Level of surgeon

The level of surgeon showed no significance with regard to overall shunt failure (*p* = *0.091*). Junior registrars placed 284 shunts, senior registrars 141 and consultants 69. Consultants had a slightly higher percentage of revision shunts placed (57.9%), as opposed to senior and junior registrars (51% and 49.6%, respectively), but this was not statistically significant.

### Shunt system

OSV II, distal slit valves, and AIS catheters were the most frequently used shunt systems at RCWMCH. Table 3 illustrates the frequency of shunts used in the index and revision groups.

**Table 3:** Shunt systems used at RCWMCH

**Shunt system**	**Index**	**Revision**	**Total**	**%**
OSV II	88	134	222	44.9
Distal slit valve	97	43	140	28.3
AIS (Bactiseal^®^)	36	54	90	18.3
Other (Miethke^®^ gravitational valve/ Integra^®^ Essential☐ valve, Atlas valve, non-specified shunt systems)	20	22	42	8.5
Total	241	253	494	

**Fig. 4:**
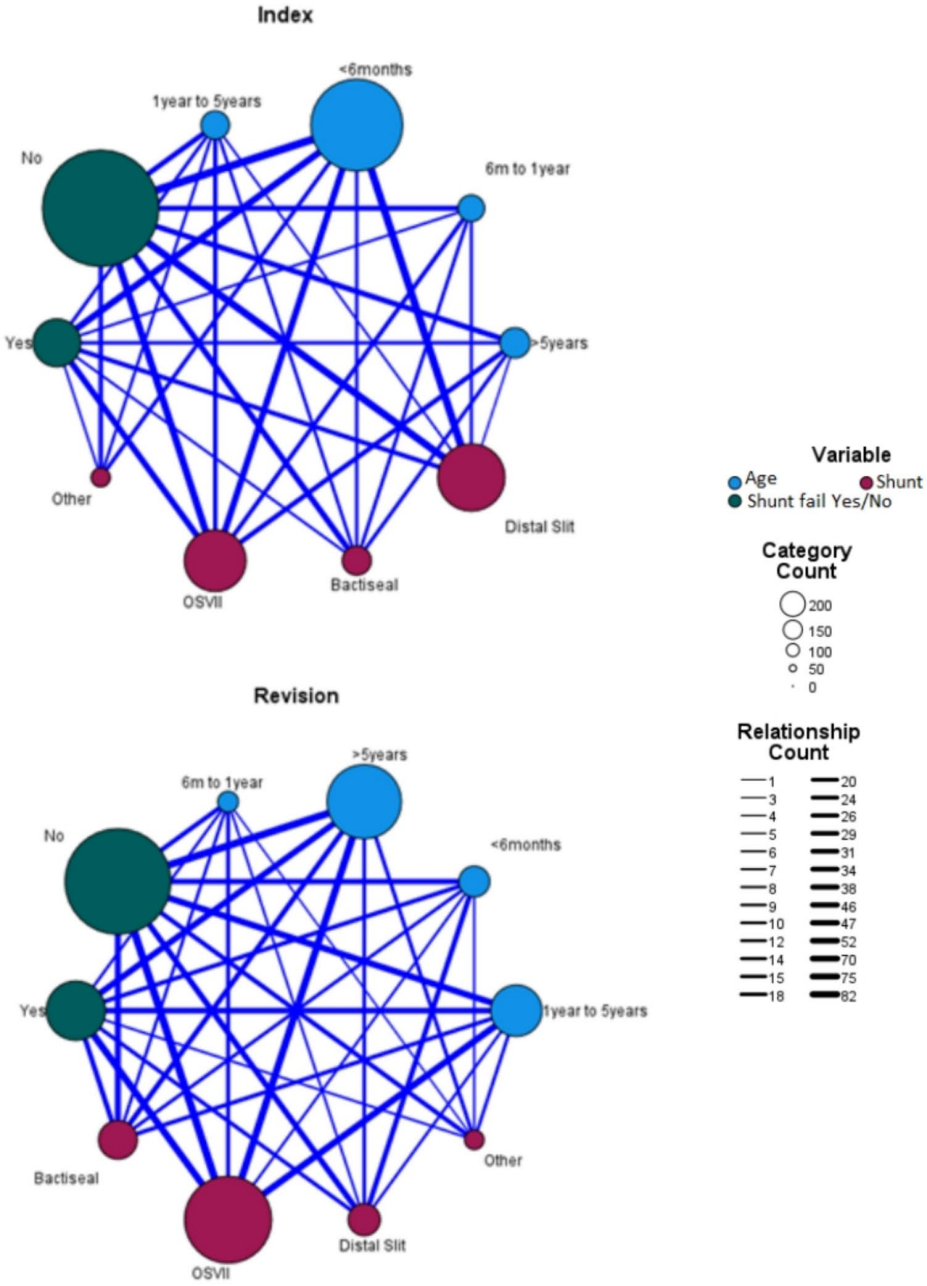
Relationship map illustrating the relationship between shunt systems used, the ages groups and the number of failures for each subgroup. Both index and revision groups are indicated. Circle size and line thickness dictate the number of each variable in the connection. The key for the relationship map is indicated on the side

Figure 4 above illustrates that distal slit valves were used more frequently in both index and revisions groups (33.6% and 9.5%) in patients < 6 months of age compared to the OSV II (14.1% and 2%) and AIS (3.3% and 3.6%) catheters. Over 6 months of age, OSV II catheters were used most frequently in both study groups. When comparing failure rates of distal slit valves to OSV II shunts, in patients under 6 months, there was no significance regarding shunt failure in both the index and revision groups (*p* = *0.100* and *p* = *0.100*, respectively).

There was no overall significance regarding the type of shunt used and risk of failure (*p* = *0.095* and *p* = *0.200*) for index and revision shunts, respectively.

### Shunt infections

Of the 90 antibiotic-impregnated catheters placed, 36 were in the index group and 54 in the revision group. There were no infections recorded in the index group. Distal slit shunts had a higher rate of infection as compared to OSV II in the index group, 25% vs 16.7%. OSV II shunts in the revision group had an infection rate of 22.2% compared to distal slit and AIS catheters (13.9% and 11.1%), although significance was not reached when comparing AIS catheters to the other shunt systems for shunt infection, both index and revision groups (*p* = *0.130* and *p* = *0.867*).

Regarding patient age and type of failure: In the revision group, patients under the age of 6 months had a higher rate of shunt infection (*p* ≤ 0.001). Significance was not achieved in the index group (*p* = 0.316).

### Follow-up

Patient folders were retrospectively analysed for 2-year follow-up data. Of the 340 patients included in the study group, 199 (58.5%) had follow-up data. Seventy-six (22.4%) did not follow up and a further 11 (3.2%) died before a 2-year follow-up. Fifty-four (15.9%) of the folders could not be located. After a 2-year follow-up outside the study period, 31 (9.1%) of the patients experienced shunt failure, with 26 (7.6%) requiring 1 revision and 5 (1.5%) requiring 2 shunt revisions.

## Discussion

Of the 494 shunts placed in 340 patients, the overall failure rate was 31.2%. Revision shunts failed more often than index shunts and this reached statistical significance. The majority of failures occurred due to blockage, both proximal and distal obstruction grouped together and our shunt infection rate of both index and revision shunts was 7.3%. Failure rates of the various shunt systems used were insignificant, as well as the influence of surgeon seniority or duration of surgery.

### Age and sex

Children younger than 2 years old have a higher shunt failure rate [11, 12, 16]. The median age of patients in this study was 15 months. We did not find a correlation between age and shunt failure. However, younger children who had revision shunts had a higher infection rate. This is replicated in other studies [16–18]. Reasons for this are not completely understood, and Kulkarni et al. described a less developed immune system in these young children as a possible contributing factor [9].

### Head circumference (OFC)

Children who had a large head circumference (above 3rd z-score) on the first presentation had a higher rate of shunt dysfunction — especially if larger than 60 cm. This was also found by Gathura et al*.* [19]. Most patients presenting to our unit charts above the 3rd z-score, reflecting late presentation, often *in extremis* and requiring urgent CSF shunt surgery. The larger heads tend to increase the chance of scalp pressure sores and potential for infection, although we did not specifically look at this. There was a high number where head circumference was not documented in the patient folders. This is — at least in part — due to the head circumference charts used, only recording up to 5 years of age.

### Aetiology

The aetiology of hydrocephalus varies across the world. In low- and middle-income countries, for example, post-infectious hydrocephalus and myelomeningocele-related are more common than in high-income countries. Warf and colleagues have documented that up to 60% of hydrocephalus cases in Africa are caused by infection [20, 21]. This is in stark contrast to the developed world, where premature IVH is the most common aetiology [11, 22]. In our study, post-infectious hydrocephalus, including TB meningitis (29.4%), followed by myelomeningocele (19.7%) and premature intraventricular haemorrhage (IVH) (14.1%), was the most common.

Several studies have investigated the aetiology of hydrocephalus and the risk for shunt failure. Berry et al. identified spinal dysraphism as a predictor for multiple shunt failures [23], and several reports have noted a lower shunt survival in children with premature IVH [24–27].

There was no significance found in this study regarding shunt failure and aetiology. This is consistent with reports of Piatt and Carlson [12].

### Shunt failure

Shunt failure has been extensively researched [28–30]. Our failure rate of 31.2%, compared to an analysis of hospital databases in Thailand by Limwattananon et al*.* [31], which showed failure rates of shunts inserted at 3, 6, 12 and 24 months being 11.5%, 19%, 25.2% and 31.3%, respectively. Rossi et al*.* [32] reported higher failure rates at 3 and 6 months of 24.1% and 29.9%. Drake et al*.* reported that first-time shunts placed in children failed 30% of the time in the first year [6].

The longer the follow-up period for shunted patients, the more it becomes clear that most will develop shunt failure at some point: longer follow-up periods up to 15 years have been described with only 15.5% of patients not requiring a shunt revision [33].

We demonstrated that revision shunts have an increased risk of shunt failure compared to index shunts. Several studies have identified a similar trend, with increasing failure rates with increasing number of shunt revisions [11, 34].

Blockage, followed by infection and disconnection were the three most common causes of shunt failure in the study for both index and revision groups. Blockage is the most widely reported cause of shunt failure worldwide, with rates ranging from 53.8 to 66.7% [11, 17].

The disconnection rate of 2.1% in index shunts and 5.1% in revision shunts is similar to a 10-year follow-up studying by Erol et al*.* of 2.6% [35] and lower than findings reported by Sainte-Rose et al. of 13.5% [36].

Misplacement of a shunt requires special mention: only 8.3% of malpositioned shunts functioned for longer than 1 year in this study. The high early and intermediate failure rate does warrant further investigation and consideration of early revision if misplacement is noted on follow-up imaging. Every safe measure should be taken to ensure accurate ventricular catheter placement in all patients.

### Shunt infection

Shunt infection rates have been widely studied with many instituting protocols to reduce infection rates [10, 14, 37–39]. There are certain protocols that are adhered to in the unit, such as cleaning with both alcohol-based solution and iodine solution, limiting manual manipulation, and frequent changing of surgical gloves, previously described by Faillace et al*.* [40], while placing and securing the shunt, although this was not analysed during this study. The department introduced disposable tunnellers in April 2016 and a change to the antibiotic protocol of Ceftriaxone and Cloxacillin from 3 days to 24 h of Cefazolin postoperatively for shunt procedures, was instituted. Limited theatre thoroughfare is enforced while shunt surgery is ongoing.

The overall shunt infection rate in this study was 7.3%. This compares well to figures reported by units around Sub-Saharan Africa of 9.1% and 9.7%, as well as units around the USA and the rest of the world, where reported figures range from 2 to 19% [17, 19, 24, 34].

The recently published BASICS trial from Mallucci et al. in 2019, as well as papers from Sciubba et al. and a meta-analysis from Klimo et al., demonstrated a significant benefit of using antibiotic-impregnated shunts (AIS) to reduce the incidence of shunt infection [41–43]. This study did not show significance regarding the use of AIS shunts and a reduction in shunt infection, although there were lower rates of infection in the AIS group compared to the distal slit valve and OSVII group. As noted, there were a greater number of AIS catheters placed in the revision group and when comparing the different shunts in this group, there was still no significance in the rate of shunt infection. It is important to note that AIS was not placed randomly; therefore, the group may be biased. We prefer to place AIS catheters in patients in whom there is a concern of future shunt infection or in patients who had presented with shunt infection, but this subgroup was not investigated in this study but would be of interest to note in future studies.

### Surgeon influence

A 1979 paper by George et al*.* reported lower rates of infection in cases done by more experienced surgeons. Similarly, Choux and Hale reported similar findings regarding surgeon experience [39, 44, 45]. A Cochrane review in 2003 demonstrated a slightly higher failure risk for lower-volume surgeons compared to their higher-volume colleagues [46]. The findings in this study did not show a significant difference in shunt failure when comparing surgeons at different levels in their training, which mirrors the findings in, The Shunt Design Trial by Kestle et al. [13]. However, shunt complexity and risk was not controlled for, and there are likely reasons for why an operation required more experienced surgeons, which likely biases the groups. Not all of these reasons are recorded here. As previously mentioned, there is a higher failure rate in patients undergoing revision shunts, and consultants are more frequently involved in the more complex revision cases in the unit. These cases tend to have higher failure rates and this may be contributing to the lack of significance found between the groups of surgeons.

### Surgery duration and time of day

As a general principle, based on the paper by Choux et al., attempts are made to keep operative time below 50 min in the unit [39]. There was a trend toward the significance of increased rates of failure in the index group with surgeries lasting more than 50 min. Reasons could be longer exposure of the shunt system to the atmosphere or increased ‘fiddle factor’ and handling of the shunt system, both with the theoretical increased risk for infection. Longer procedures may also imply more complicated operations, such as for difficult peritoneal access in patients with previous abdominal scarring.

Revision surgeries tended to take longer; a trend commonly seen with any revision surgery.

The elective or emergency nature of cases theoretically may influence shunt outcomes. There was no association between the time of day that the shunt was inserted, although we did not control for bias: more complicated shunt surgeries are generally done during the daytime and consultant involvement is preferred.

### Endoscope

The use of an endoscope to assist with shunt insertion has been described to increase the risk of shunt infection and subsequent failure [8, 45]. Complex hydrocephalus may necessitate an endoscope. The use of the endoscope for ETVs and intraventricular biopsies was also included if the patient had to have a ventriculoperitoneal shunt inserted during their admission. There was no associated increase in shunt infection or failure noted in this study group, although the sample size was small. Given the relatively low number of endoscopic-assisted shunts placed in the study period, it may be helpful in future studies to include a large sample size. It may also be important to note that these procedures will be done by a consultant. This may affect the outcome.

### CSF protein and red cell concentration

There was no association found with CSF protein or red blood cell level and the rate of shunt failure, as described in other studies [47, 48]. Protein and cell count may have influenced surgeons’ choice of shunt, though, which may influence the outcome. In the past, surgeons in the unit would have opted for a distal slit valve shunt in these cases. This was not examined in this study but could be a future subject to investigate.

### Follow up

A major limitation of every retrospective review, especially in a folder-based system such as ours, is the loss of data. At the time of this study, 15.9% of the folders could not be retrieved. Furthermore, loss to follow-up is common in a mobile population — several of the patients in our system fall outside of our main referral region. This study was limited to our unit, and a national database does not exist; hence, no conclusions can be drawn from the 22.4% of patients who did not follow-up, regarding shunt failure. These patients could have potentially returned to their home province and required further shunt surgery, demised in the community, or may be living without any shunt issues. This is unfortunately a high rate of attrition common in environments such as ours.

### Limitations of study

This study is a retrospective study and results are subject to bias, some of which have already been described. Multiple factors may have contributed to the differences found between the two groups analysed, not all of which are documented. Conversely, identification of certain risk factors may have required a larger sample size. The data collected were based on available clinical data and as mentioned, the cause of shunt failure was left open to interpretation by the operating surgeon. As certain patients required multiple revision surgeries, it is possible that this patient subgroup may skew the overall failure rates but is important in the overall picture of shunt failure. Various factors potentially associated with outcomes are skewed by bias in different groups: for example, the experience of the surgeon, timing of surgery and choice of shunt type. Prospective data collection, or better yet, randomisation where possible, might yield better results. Finally, missing data may influence the results and the true 2-year failure rate could be different than the quoted figure.

## Conclusion

VP shunt failure is commonly encountered by neurosurgeons around the world. Our universal desire to improve shunt survival and investigate potential causes of shunt failure is clear. This study documents the institution’s overall shunt failure rate based on existing data, and as such forms the foundation for quality improvement initiatives. The overall failure rate was 31.2% and the shunt infection rate was 7.3%. Revision cases have higher failure rates. There was a considerable number of children presenting with grossly increased head circumferences, a problem faced by the developing well at large. The early failure of misplaced ventricular catheters does support the practice of routine early post-operative imaging and a low threshold to revise malpositioned catheters. By remaining critical of one’s own shunt outcomes as well as trying to improve access to care and timing of intervention can we endeavour to improve a lot of these desperate children.

## Data Availability

No datasets were generated or analysed during the current study.

## References

[1] Dewan MC, Rattani A, Mekary R, Glancz LJ, Yunusa I, Baticulon RE et al (2019) Global hydrocephalus epidemiology and incidence: systematic review and meta-analysis. J Neurosurg 130(4):1065–1079 29701543 10.3171/2017.10.JNS17439

[2] Nawaz S, Hayat F, Khan S, Rehman S, Sardar N, Aman S (2019) Causes of hydrocephalus and complications of VP shunt in pediatric population. Gomal Journal of Medical Sciences 16(4):97–100 10.46903/gjms/16.04.1694

[3] Muir RT, Wang S, Warf BC (2016) Global surgery for pediatric hydrocephalus in the developing world: a review of the history, challenges, and future directions. Neurosurg Focus 41(5):E11. 10.3171/2016.7.FOCUS16273. PMID: 27798988 27798988 10.3171/2016.7.FOCUS16273

[4] Dewan MC, Rattani A, Fieggen G, Arraez MA, Servadei F, Boop FA et al (2018) Global neurosurgery: the current capacity and deficit in the provision of essential neurosurgical care. Executive Summary of the Global Neurosurgery Initiative at the Program in Global Surgery and Social Change. J Neurosurg 1–10 10.3171/2017.11.JNS171500 29701548

[5] Mock CN, Donkor P, Gawande A, Jamison DT, Kruk ME, Debas HT (2015) Essential surgery: key messages from disease control priorities, 3rd edition. Lancet 385(9983):2209–19 25662414 10.1016/S0140-6736(15)60091-5 PMC7004823

[6] Drake JM, Kestle J, Boop F, Cochrane D, Haines S, Sainte-Rose C et al (1996) Rationale and methodology of the multicenter pediatric cerebrospinal fluid shunt design trial. Childs Nerv Syst 12(8):434–447 8891361 10.1007/BF00261620

[7] Kulkarni AV, Riva-Cambrin J, Butler J, Browd SR, Drake JM, Holubkov R et al (2013) Outcomes of CSF shunting in children: comparison of Hydrocephalus Clinical Research Network cohort with historical controls: clinical article. J Neurosurg Pediatr 12(4):334 23909616 10.3171/2013.7.PEDS12637

[8] Riva-Cambrin J, Kestle JRW, Holubkov R, Butler J, Kulkarni AV, Drake J et al (2016) Risk factors for shunt malfunction in pediatric hydrocephalus: a multicenter prospective cohort study. J Neurosurg Pediatr 17(4):382 26636251 10.3171/2015.6.PEDS14670

[9] Kulkarni AV, Drake JM, Lamberti-Pasculli M (2001) Cerebrospinal fluid shunt infection: a prospective study of risk factors. J Neurosurg 94(2):195 11213954 10.3171/jns.2001.94.2.0195

[10] Sarmey N, Kshettry V, Shriver M, Habboub G, Machado A, Weil R (2015) Evidence-based interventions to reduce shunt infections: a systematic review. Childs Nerv Syst 31(4):541–549 25686893 10.1007/s00381-015-2637-2

[11] Tuli S, Drake J, Lawless J, Wigg M, Lamberti-Pasculli M (2000) Risk factors for repeated cerebrospinal shunt failures in pediatric patients with hydrocephalus. J Neurosurg 92(1):31–38 10616079 10.3171/jns.2000.92.1.0031

[12] Piatt JH Jr, Carlson CV (1993) A search for determinants of cerebrospinal fluid shunt survival: retrospective analysis of a 14-year institutional experience. Pediatr Neurosurg 19:233–242 8398847 10.1159/000120738

[13] Kestle J, Milner R, Drake J (1999) The shunt design trial: variation in surgical experience did not influence shunt survival. Pediatr Neurosurg 30(6):283–287 10494053 10.1159/000028812

[14] Kestle J, Riva-Cambrin J, Wellons J, Kulkarni A, Whitehead W, Walker M et al (2011) A standardized protocol to reduce cerebrospinal fluid shunt infection: the Hydrocephalus Clinical Research Network Quality Improvement Initiative Clinical article. J Neurosurg Pediatr 8(1):22–29 21721884 10.3171/2011.4.PEDS10551 PMC3153415

[15] Shannon C, Acakpo-Satchivi L, Kirby R, Franklin F, Wellons J (2012) Ventriculoperitoneal shunt failure: an institutional review of 2-year survival rates. Childs Nerv Syst 28(12):2093–2099 22706983 10.1007/s00381-012-1830-9

[16] Liptak GS, McDonald JV (2004) Ventriculoperitoneal shunts in children: factors affecting shunt survival. Pediatr Neurosurg 12(6):289–293 10.1159/000120268 3870651

[17] Mwachaka PM, Obonyo NG, Mutiso BK, Ranketi S, Mwang’ombe N (2010) Ventriculoperitoneal shunt complications: a three-year retrospective study in a Kenyan national teaching and referral hospital. Pediatr Neurosurg 46(1):1–5 20453556 10.1159/000314050

[18] Simon TD, Whitlock KB, Riva-Cambrin J, Kestle JRW, Rosenfeld M, Dean JM et al (2012) Revision surgeries are associated with significant increased risk of subsequent cerebrospinal fluid shunt infection. Pediatr Infect Dis J 31(6):551–556 22333701 10.1097/INF.0b013e31824da5bd PMC3356497

[19] Gathura E, Poenaru D, Bransford R, Albright AL (2010) Outcomes of ventriculoperitoneal shunt insertion in Sub-Saharan Africa: clinical article. J Neurosurg Pediatr 6(4):329–335 20887104 10.3171/2010.7.PEDS09543

[20] Warf BC (2005) Hydrocephalus in Uganda: the predominance of infectious origin and primary management with endoscopic third ventriculostomy. J Neurosurg 102(1):1–15 16206728 10.3171/ped.2005.102.1.0001

[21] Kulkarni AV, Warf BC, Drake JM, Mallucci CL, Sgouros S, Constantini S (2010) Surgery for hydrocephalus in sub-Saharan Africa versus developed nations: a risk-adjusted comparison of outcome. Childs Nerv Syst 26(12):1711–1717 20552204 10.1007/s00381-010-1195-x

[22] Abdullah J, Naing NN (2001) Hydrocephalic children presenting to a Malaysian community-based university hospital over an 8-year period. Pediatr Neurosurg 34(1):13–19 11275782 10.1159/000055987

[23] Berry JG, Hall MA, Sharma V, Goumnerova L, Slonim AD, Shah SS (2008) A multi-institutional, 5-year analysis of initial and multiple ventricular shunt revisions in children. Neurosurgery 62(2):445–453 18382323 10.1227/01.neu.0000316012.20797.04

[24] Hauptman JS, Kestle J, Riva-Cambrin J, Kulkarni AV, Browd SR, Rozzelle CJ et al (2021) Predictors of fast and ultrafast shunt failure in pediatric hydrocephalus: a Hydrocephalus Clinical Research Network study. J Neurosurg Pediatr 27(3):277–286 10.3171/2020.7.PEDS20111 33338993

[25] Albright AL, Pollack IF, Adelson PD, Solot JJ (1999) Outcome data and analysis in pediatric neurosurgery. Neurosurgery 45(1):101–106 10414572 10.1097/00006123-199907000-00025

[26] Cozzens JW, Chandler JP (1997) Increased risk of distal ventriculoperitoneal shunt obstruction associated with slit valves or distal slits in the peritoneal catheter. J Neurosurg 87(5):682–686 9347975 10.3171/jns.1997.87.5.0682

[27] Khan F, Shamim MS, Rehman A, Bari ME (2013) Analysis of factors affecting ventriculoperitoneal shunt survival in pediatric patients. Childs Nerv Syst 29(5):791–802 23296321 10.1007/s00381-012-2004-5

[28] Shannon CN, Carr KR, Tomycz L, Wellons JC, Tulipan N (2015) Time to first shunt failure in pediatric patients over 1 year old: a 10-year retrospective study. Pediatr Neurosurg 49(6):353–359 10.1159/000369031 25471222

[29] Al-Tamimi YZ, Sinha P, Chumas PD, Crimmins D, Drake J, Kestle J et al (2014) Ventriculoperitoneal shunt 30-day failure rate: a retrospective international cohort study. Neurosurgery 74(1):29–34 24089046 10.1227/NEU.0000000000000196

[30] Anderson IA, Saukila LF, Robins JMW, Akhunbay-Fudge CY, Goodden JR, Tyagi AK et al (2019) Factors associated with 30-day ventriculoperitoneal shunt failure in pediatric and adult patients. J Neurosurg 130(1):145–153 29521592 10.3171/2017.8.JNS17399

[31] Limwattananon P, Kitkhuandee A (2021) Ventriculoperitoneal shunt failure in pediatric patients: an analysis of a national hospitalization database in Thailand. J Neurosurg Pediatr 28(2):128–138 34087796 10.3171/2021.1.PEDS20718

[32] Rossi NB, Khan NR, Jones TL, Lepard J, McAbee JH, Mph PKMD (2016) Predicting shunt failure in children: should the global shunt revision rate be a quality measure? J Neurosurg Pediatr 17(3):249–259 26544083 10.3171/2015.5.PEDS15118

[33] Stone JJ, Walker CT, Jacobson M, Phillips V, Silberstein HJ (2013) Revision rate of pediatric ventriculoperitoneal shunts after 15 years: clinical article. J Neurosurg Pediatr 11(1):15–19 23101557 10.3171/2012.9.PEDS1298

[34] McGirt MJ, Leveque J-C, Wellons Iii JC, Villavicencio AT, Hopkins JS, Fuchs HE et al (2002) Cerebrospinal fluid shunt survival and etiology of failures: a seven-year institutional experience. Pediatr Neurosurg 36(5):248–255 12053043 10.1159/000058428

[35] Erol FS, Ozturk S, Akgun B, Kaplan M (2017) Ventriculoperitoneal shunt malfunction caused by fractures and disconnections over 10 years of follow-up. Childs Nerv Syst 33(3):475–481 28097382 10.1007/s00381-017-3342-0

[36] Sainte-Rose C, Piatt JH, Renier D, Pierre-Kahn A, Hirsch JF, Hoffman HJ et al (2004) Mechanical complications in shunts. Pediatr Neurosurg 17(1):2–9 10.1159/000120557 1811706

[37] Choksey MS, Malik IA (2004) Zero tolerance to shunt infections: can it be achieved? J Neurol Neurosurg Psychiatry 75(1):87–91 14707314 PMC1757446

[38] Pirotte BJM, Lubansu A, Bruneau M, Loqa C, Van Cutsem N, Brotchi J (2007) Sterile surgical technique for shunt placement reduces the shunt infection rate in children: preliminary analysis of a prospective protocol in 115 consecutive procedures. Childs Nerv Syst 23(11):1251–1261 17705062 10.1007/s00381-007-0415-5

[39] Choux M, Genitori L, Lang D, Lena G (1992) Shunt implantation: reducing the incidence of shunt infection. J Neurosurg 77(6):875–880 1432129 10.3171/jns.1992.77.6.0875

[40] Faillace WJ (1995) A no-touch technique protocol to diminish cerebrospinal fluid shunt infection. Surg Neurol 43(4):344–350 7792703 10.1016/0090-3019(95)80060-T

[41] Mallucci CL, Jenkinson MD, Conroy EJ, Hartley JC, Brown M, Dalton J et al (2019) Antibiotic or silver versus standard ventriculoperitoneal shunts (BASICS): a multicentre, single-blinded, randomised trial and economic evaluation. Lancet (British edition) 394(10208):1530–1539 10.1016/S0140-6736(19)31603-4 PMC6999649 31522843

[42] Sciubba DM, Stuart RM, McGirt MJ, Woodworth GF, Samdani A, Carson B et al (2005) Effect of antibiotic-impregnated shunt catheters in decreasing the incidence of shunt infection in the treatment of hydrocephalus. J Neurosurg 103(2):131–136 16370278 10.3171/ped.2005.103.2.0131

[43] Klimo P Jr, Thompson CJ, Ragel BT, Boop FA (2011) Antibiotic-impregnated shunt systems versus standard shunt systems: a meta- and cost-savings analysis: Clinical article. J Neurosurg Pediatr 8(6):600–612 22132919 10.3171/2011.8.PEDS11346

[44] George R, Leibrock L, Epstein M (1979) Long-term analysis of cerebrospinal fluid shunt infections. A 25-year experience. J Neurosurg 51(6):804–11 501424 10.3171/jns.1979.51.6.0804

[45] Hale AT, Riva-Cambrin J, Wellons JC, Jackson EM, Kestle JRW, Naftel RP et al (2021) Machine learning predicts risk of cerebrospinal fluid shunt failure in children: a study from the hydrocephalus clinical research network. Childs Nerv Syst 37(5):1485–1494 33515058 10.1007/s00381-021-05061-7

[46] Cochrane DD, Kestle JRW (2003) The influence of surgical operative experience on the duration of first ventriculoperitoneal shunt function and infection. Pediatr Neurosurg 38(6):295–301 12759508 10.1159/000070413

[47] Cheatle JT, Bowder AN, Tefft JL, Agrawal SK, Hellbusch LC (2015) Effect of protein concentration on the flow of cerebrospinal fluid through shunt tubing. Neurosurgery 77(6):972–978 26270195 10.1227/NEU.0000000000000956

[48] Brydon HL, Hayward R, Harkness W, Bayston R (1996) Does the cerebrospinal fluid protein concentration increase the risk of shunt complications? Br J Neurosurg 10(3):267–274 8799537 10.1080/02688699650040124

